# Restoration of mGluR6 Localization Following AAV-Mediated Delivery in a Mouse Model of Congenital Stationary Night Blindness

**DOI:** 10.1167/iovs.62.3.24

**Published:** 2021-03-17

**Authors:** Juliette Varin, Nassima Bouzidi, Miguel Miranda De Sousa Dias, Thomas Pugliese, Christelle Michiels, Camille Robert, Melissa Desrosiers, José-Alain Sahel, Isabelle Audo, Deniz Dalkara, Christina Zeitz

**Affiliations:** 1 Sorbonne Université, INSERM, CNRS, Institut de la Vision, Paris, France; 2 CHNO des Quinze-Vingts, DHU Sight Restore, INSERM-DGOS CIC 1423, Paris, France; 3 Fondation Ophtalmologique Adolphe de Rothschild, Paris, France; 4 Academie des Sciences, Institut de France, Paris, France; 5 Department of Ophthalmology, The University of Pittsburgh School of Medicine, Pittsburgh, Pennsylvania, United States; 6 Institute of Ophthalmology, University College of London, London, United Kingdom

**Keywords:** CSNB, gene therapy, bipolar cells, mGluR6

## Abstract

**Purpose:**

Complete congenital stationary night blindness (cCSNB) is an incurable inherited retinal disorder characterized by an ON-bipolar cell (ON-BC) defect. *GRM6* mutations are the third most prevalent cause of cCSNB. The *Grm6^−^^/^^−^* mouse model mimics the human phenotype, showing no b-wave in the electroretinogram (ERG) and a loss of mGluR6 and other proteins of the same cascade at the outer plexiform layer (OPL). Our aim was to restore protein localization and function in *Grm6^−^^/^^−^* adult mice targeting specifically ON-BCs or the whole retina.

**Methods:**

Adeno-associated virus-encoding *Grm6* under two different promoters (GRM6-*Grm6* and CAG-*Grm6*) were injected intravitreally in P15 *Grm6^−^*^/^*^−^* mice. ERG recordings at 2 and 4 months were performed in *Grm6^+/+^*, untreated and treated *Grm6^−^^/^^−^* mice. Similarly, immunolocalization studies were performed on retinal slices before or after treatment using antibodies against mGluR6, TRPM1, GPR179, RGS7, RGS11, Gβ5, and dystrophin.

**Results:**

Following treatment, mGluR6 was localized to the dendritic tips of ON-BCs when expressed with either promoter. The relocalization efficiency in mGluR6-transduced retinas at the OPL was 2.5% versus 11% when the GRM6-*Grm6* and CAG-*Grm6* were used, respectively. Albeit no functional rescue was seen in ERGs, relocalization of TRPM1, GPR179, and Gβ5 was also noted using both constructs. The restoration of the localization of RGS7, RGS11, and dystrophin was more obvious in retinas treated with GRM6-*Grm6* than in retinas treated with CAG-*Grm6*.

**Conclusions:**

Our findings show the potential of treating cCSNB with *GRM6* mutations; however, it appears that the transduction rate must be improved to restore visual function.

Congenital stationary night blindness (CSNB) is a non-progressive retinal disorder.^1^ Patients suffering from this disease often present an impairment of night vision, delayed light-to-dark adaptation, and difficulty with properly sensing contrasts in dim-light conditions. It is also often associated with high myopia, strabismus, and nystagmus.^2^ Considering the retinal response to the full-field electroretinogram (ERG), CSNB has been divided in two types: Riggs CSNB, which is associated with a rod-photoreceptor defect, and Schubert–Bornschein CSNB,^1^ in which the underlying defect relies on the signal transmission from photoreceptors to bipolar cells. The Schubert–Bornschein type of CSNB can be classified as incomplete CSNB or complete CSNB (cCSNB).^3^ We focused on the latter type in this study. Patients with cCSNB show an electronegative ERG waveform, with normal a-wave amplitudes and severely diminished or absent b-waves under scotopic conditions, thus referring to it as complete. Photopic responses are less altered.^2^ More specifically, ON-responses are affected but OFF-responses remain globally normal.^2^ This ERG profile is consistent with a transmission defect between photoreceptors and ON-bipolar cells (ON-BCs). cCSNB is due to mutations in *NYX*,^4^^,^^5^ *TRPM1*,^6^^–^^8^ *GRM6*,^9^^,^^10^ *GPR179*,^11^^,^^12^ and *LRIT3*.^13^ All code for proteins involved in the signaling cascade at the photoreceptor to ON-BC synapse.

Mutations in *GRM6* are the third most prevalent cause of cCSNB.^2^ *GRM6* codes for a metabotropic receptor, (mGluR6) that mediates glutamate synaptic transmission between photoreceptors and ON-BCs. In the retina, metabotropic glutamate receptor 6 (mGluR6) is exclusively localized in ON-BCs.^14^ In the absence of light, glutamate released at the synaptic cleft activates mGluR6, which initiates the ON-BC signaling cascade^15^ leading to closure of the cation channel transient receptor potential melastatin 1 (TRPM1).^16^ In contrast, in response to light, less glutamate binds to mGluR6, leading to opening of the TRPM1 channel.^17^ Subsequently, ON-BCs are depolarized, leading to the b-wave, which is severely reduced or absent in cCSNB. Five mouse models with a *Grm6* defect have been described: *Grm6^tm1Nak^* (referred to here as *Grm6^−^^/^^−^*), *nob3*, *nob4*, *nob7*, and *nob8*.^18^^–^^22^ Of those, four (including *Grm6^−^^/^^−^*) show a similar ERG phenotype as patients with cCSNB under scotopic conditions—namely, an absent b-wave, whereas the a-wave appears normal. Furthermore, in these four models, mGluR6 is absent.

It has also been suggested that mGluR6 is important for the proper localization of other proteins of the cascade, such as regulator G protein signaling (RGS) proteins (RGS7, RGS11, Gβ5, and R9AP),^23^^,^^24^ and TRPM1^23^ at the dendritic tips of ON-BCs. The absence of TRPM1 at the dendritic tips of ON-BCs renders this channel nonfunctional.^23^ The interdependence of G protein-coupled receptor 179 (GPR179) on mGluR6, important for the targeting and/or maintenance of RGS proteins,^25^ was found to be model and condition dependent.^26^^,^^27^ Albeit the gross morphology of the retina in patients and mice lacking mGluR6 is not affected, detailed clinical examinations and morphological studies have detected differences compared to unaffected retinas. Indeed, three patients harboring mutations in *GRM6* exhibited reduced retinal thickness in the extrafoveal region, as revealed by spectral-domain optical coherence tomography measurements.^28^ Furthermore, in mice lacking *Grm6*, invaginating dendrites of rod BCs are larger and often contain ectopic ribbons, whereas the number of invaginating dendrites of cone ON-BCs and ribbons decreases at the cone pedicles in the *Grm6^−^^/^^−^* mouse model.^29^ Tummala and colleagues^30^ showed that mGluR6 has also a role presynaptically, as several presynaptic matrix-associated proteins such as dystrophin are reduced at the photoreceptor-to-BC synapse in the same *Grm6^−^^/^^−^* model.

To date, no treatment is available for cCSNB. A gene replacement strategy might be a suitable approach treating cCSNB, as it represents a stationary nondegenerative disorder, and the genetics of this disorder are well characterized.^2^ Two different approaches targeting two genes involved in cCSNB, *Nyx* and *Lrit3*, resulted in partial restoration of function upon protein relocalization in mouse models.^31^^,^^32^ However, this partial rescue was mainly obtained at a very young age (postnatal day 2 [P2] or 5 [P5]) and only under scotopic conditions. These two strategies aimed at treating cCSNB show the challenges to obtaining functional rescue in adult cCSNB mice. Our study aimed to restore GluR6 localization, along with its missing partners and ERG phenotype, by treating adult P15 *Grm6^−^^/^^−^*^18^ mice with an adeno-associated virus (AAV)-mediated intravitreal delivery of the transgene. In order to compare their efficacy, two different promoters were used: (1) a *Grm6*-200bp/SV40 promoter that specifically targets ON-BCs (referred to here as the *GRM6* promoter) and (2) a cytomegalovirus (CMV) enhancer fused to the chicken β-actin promoter (CAG) promoter driving ubiquitous expression of the transgene. Protein localization, and functional rescue were investigated.

## Materials and Methods

### Ethical Statement

All animal procedures were performed according to Council Directive 2010/63EU of the European Parliament and the Council of September 22, 2010, on the protection of animals used for scientific purposes; National Institutes of Health guidelines; and the ARVO Statement for the Use of Animals in Ophthalmic and Vision Research. The study was approved by the French Minister of National Superior Education and Research (authorization delivered on January 21, 2019; APAFIS: #1132-201812181048827 v2).

### Intravitreal Injections

Two different constructs were designed using two different promoters to rescue the phenotype in the *Grm6^−^^/^^−^* mouse model. To drive the expression of the transgene specifically to ON-BCs, the *GRM6* promoter was used.^33^ To drive ubiquitous expression of the transgene throughout the retina, the CAG promoter was used.^34^ These two constructs were encapsidated in the AAV2-7m8 serotype^34^ and are referred to here as GRM6-*Grm6* and CAG-*Grm6*, respectively. Mice were anesthetized by isoflurane inhalation (5% in oxygen for induction and 2% for maintenance). Intravitreal injections were performed at postnatal day 15. Pupils were dilated (0.5% mydriaticum), and a 33-gauge needle was passed through the sclera at the ora serrata level. A total of 1 µL of viral stock solution at maximum concentrations of 1.6 × 10^13^ viral genomes (vg)/mL (CAG-*Grm*6 construct) and 5.8 × 10^13^ vg/mL (GRM6-*Grm6* construct) was injected directly in the vitreous cavity of eight *Grm6^−^^/^^−^* mice for each promoter. Viral vectors were produced as described in Macé et al.^33^

### Immunolocalization Studies

Animals were sacrificed by CO_2_ inhalation followed by cervical dislocation. Eyes were removed and dissected to keep the posterior part of the eyes, which were then fixed in ice-cold 4% paraformaldehyde for 20 minutes. Subsequently, eyecups were washed in ice-cold PBS and cryoprotected by increasing concentrations of sucrose (ranging from 10% to 30%) in water and 0.24-M phosphate buffer for 1 hour at 4°C for the 10% sucrose and 20% sucrose solutions and overnight at 4°C under agitation for the 30% sucrose solution. The eyecups were then embedded in 7.5% gelatin/10% sucrose; the blocks were frozen at –40°C in isopentane and kept at –80°C until cutting. Sections of 12-µm thickness were generated using a cryostat (Microm HM 560; Thermo Fisher Scientific, Waltham, MA, USA) and mounted on glass slides (Superfrost Plus; Thermo Fisher Scientific). Mouse retinal sections were blocked for 1 hour at room temperature in PBS, 1×; 10% donkey serum (v/v); and 0.3% Triton X-100. Primary antibodies and the dilutions used were sheep anti-TRPM1 (1:500; Cao et al.^35^), guinea pig anti-mGluR6 (AP20134SU-N, 1:15000; Acris, Herford, Germany), rabbit anti-Gβ5 (C16068, 1:500; Antibodies Online, Limerick, PA, USA), goat anti-RGS11 (sc-9725, 1:300; Santa-Cruz Biotechnology, Dallas, TX, USA), rabbit anti-RGS7 (1:100; Cao et al.^36^), mouse anti-GPR179 (AB0887-YOM, 1:200; PrimmBiotech, Cambridge, MA, USA), and H4 anti-dystrophin (1:1000; Vacca et al.^37^). The sections were incubated with primary antibodies diluted in PBS, 1×; 2% donkey serum; and 0.1% Triton X-100 for 1 hour at room temperature. After washes with PBS, 1×, and 0.1% Triton X-100, the sections were incubated with anti-human, anti-guinea pig, anti-goat, anti-rabbit, or anti-mouse secondary antibodies coupled with Alexa Fluor 488, Alexa Fluor 594, or Cy3 (Jackson ImmunoResearch Laboratories, West Grove, PA, USA) along with 4′,6-diamidino-2-phenylindole, all used at 1:1000, for 0.5 hour at room temperature. Subsequently, the sections were coverslipped with mounting medium (Mowiol; Millipore Sigma, Burlington, MA, USA). Fluorescence images of retinal sections were acquired with a confocal microscope (FV1000; Olympus, Tokyo, Japan). Images for figures were handled with Image J (National Institutes of Health, Bethesda, MD, USA). The percentage of outer plexiform layer (OPL) presenting any mGluR6 staining was automatically calculated using Image J. After selection of the OPL area, the ratio of red staining representing mGluR6 compared to green staining representing PKCα was determined.

### ERG Recordings

Full-field ERG recordings were performed in accordance with the description in Neuillé et al.^38^ All scotopic ERG responses were recorded first using six increasing light intensities of flashes ranging from 0.003 to 30.0 cd·s/m^2^. To ensure saturation of rod photoreceptors and the recording of cone-driven responses, a 10-minute light-adaptation step at 20 cd/m^2^ was done. All data were analyzed with Prism v.6 (GraphPad Software, La Jolla, CA, USA).

## Results

### mGluR6 Relocalized at the Dendritic Tips of ON-BCs Following Treatment

**Figure 1.**
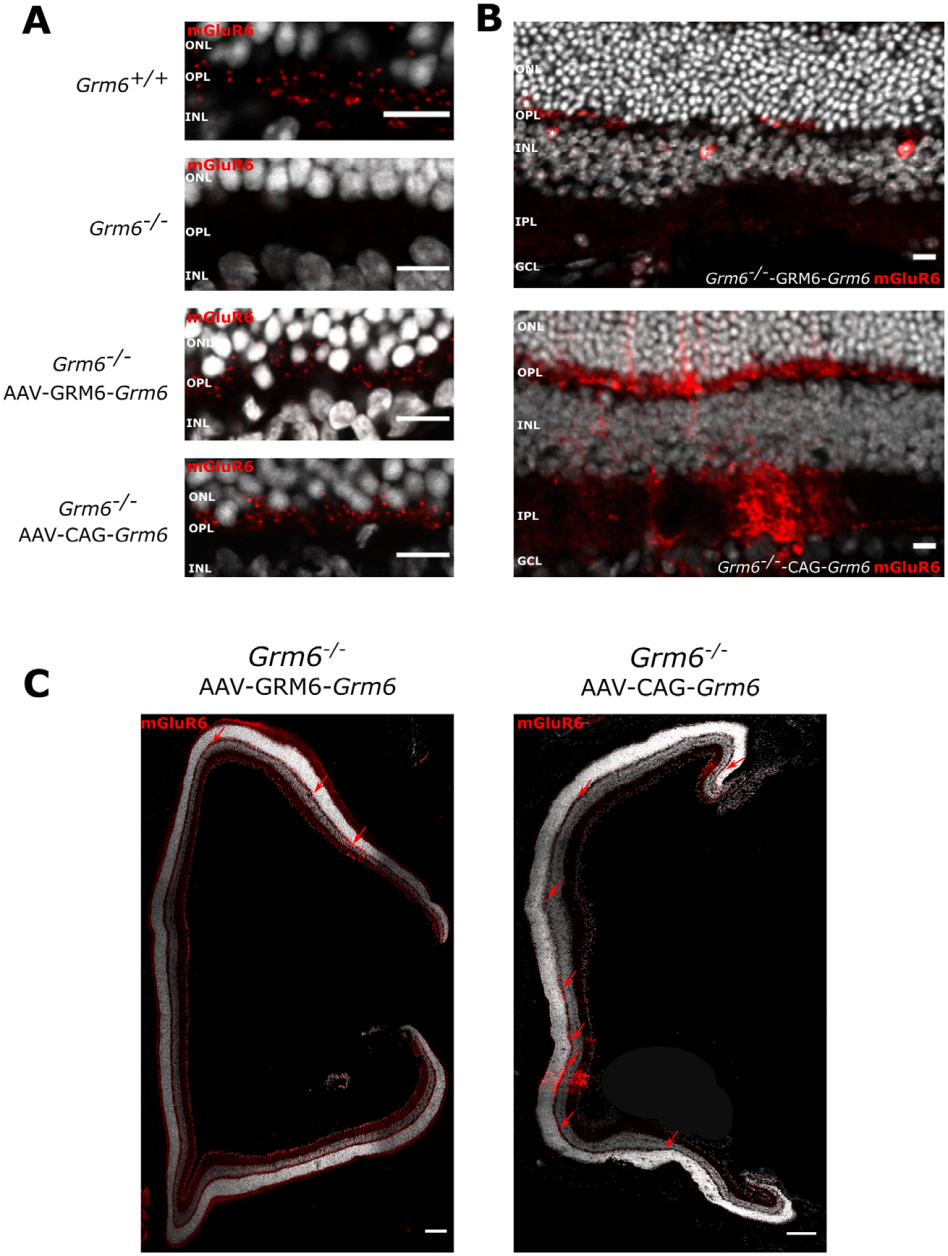
GRM6 localization after treatment. (**A**) Representative confocal images of cross-sections centered on the OPL of *Grm6^+^^/^^+^*, untreated *Grm6^−^^/^^−^*, *Grm6^−^^/^^−^*-GRM6-*Grm6*, and *Grm6^−^^/^^−^*-CAG-*Grm6* retinas stained with an antibody against mGluR6 (*red*). *Scale bar*: 10 µm. (**B**) Specific features of *Grm6^−^^/^^−^*-GRM6-*Grm6* (*top row*, stars represent putative somas of ON-BCs) and *Grm6^−^^/^^−^*-CAG-*Grm6* (*bottom row*, diffuse staining) retinas. ONL, outer nuclear layer; INL, inner nuclear layer, IPL, inner plexiform layer; GCL, ganglion cell layer. *Scale bar*: 10 µm. (**C**) Representative confocal images of cross-sections of the entire retina of mice treated with *Grm6^−^^/^^−^*-GRM6-*Grm6* or *Grm6^−^^/^^−^*-CAG-*Grm6*. *Red arrows* point to mGluR6 staining. *Scale bar*: 100 µm.

**Figure 2.**
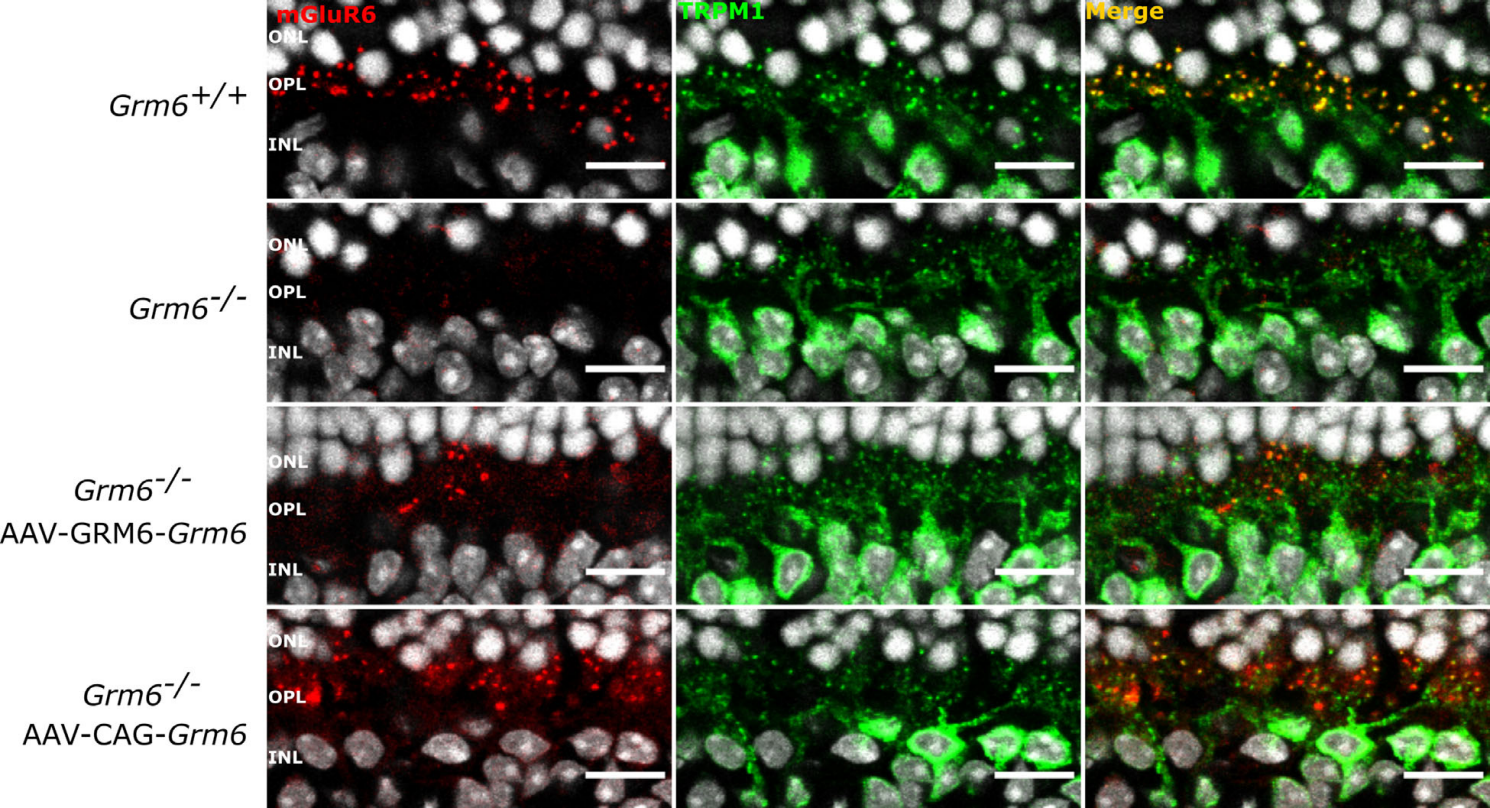
Localization of TRPM1 after treatment. Representative confocal images of cross-sections centered on the OPL of *Grm6^+/+^*, untreated *Grm6^−^^/^^−^*, *Grm6^−^^/^^−^*-GRM6-*Grm6*, and *Grm6^−^^/^^−^*-CAG-*Grm6* retinas co-stained (*yellow*, merge) with an antibody against mGluR6 (*red*) and against TRPM1 (*green*). *Scale bar*: 10 µm.

In *Grm6^−^^/^^−^* mice, mGluR6 production was completely absent in the OPL (Fig. 1A)^18^. Its localization at the dendritic tips of ON-BCs is essential to ensure proper transmission of the visual information between photoreceptors and ON-BCs. Relocalization of mGluR6 at the dendritic tips of ON-BCs 5 months following treatment was investigated through immunolocalization studies. *Grm6^−^^/^^−^*-GRM6-*Grm6* retinas treated at P15 displayed areas where mGluR6 was present in the OPL but which was absent in untreated *Grm6^−^^/^^−^* retinas (*n* = 8) (Fig. 1A). We also noticed that ON-BC bodies were sometimes stained (Fig. 1B, asterisks). However, mGluR6 staining was only present in small areas of the retina (∼2.5% of the OPL presented any mGluR6 staining) (Fig. 2C, red arrows). Similarly, *Grm6^−^^/^^−^*-CAG-*Grm6* retinas treated at P15 displayed mGluR6 staining in the OPL (Fig. 1A), which seemed to be stronger and more homogeneously distributed (Figs. 1B, 1C) compared to the *Grm6^−^^/^^−^*-GRM6-*Grm6* treated retinas (∼11% of the OPL presented any mGluR6 staining) (Fig. 1C, red arrows). In addition, for the latter construct, staining was also noted in some areas in the inner plexiform layer (IPL) (Fig. 1B). Furthermore, this mGluR6 staining seemed more diffuse in several areas (Fig. 1B). Using either CAG-*Grm6 or* GRM6-*Grm6* constructs, mGluR6 staining was solely observed at the dendritic tips of rod BCs; no staining was observed at the dendritic tips of cone ON-BCs.

### Signaling Partners of mGluR6 Were Relocalized at the Dendritic Tips of ON-BCs

**Figure 3.**
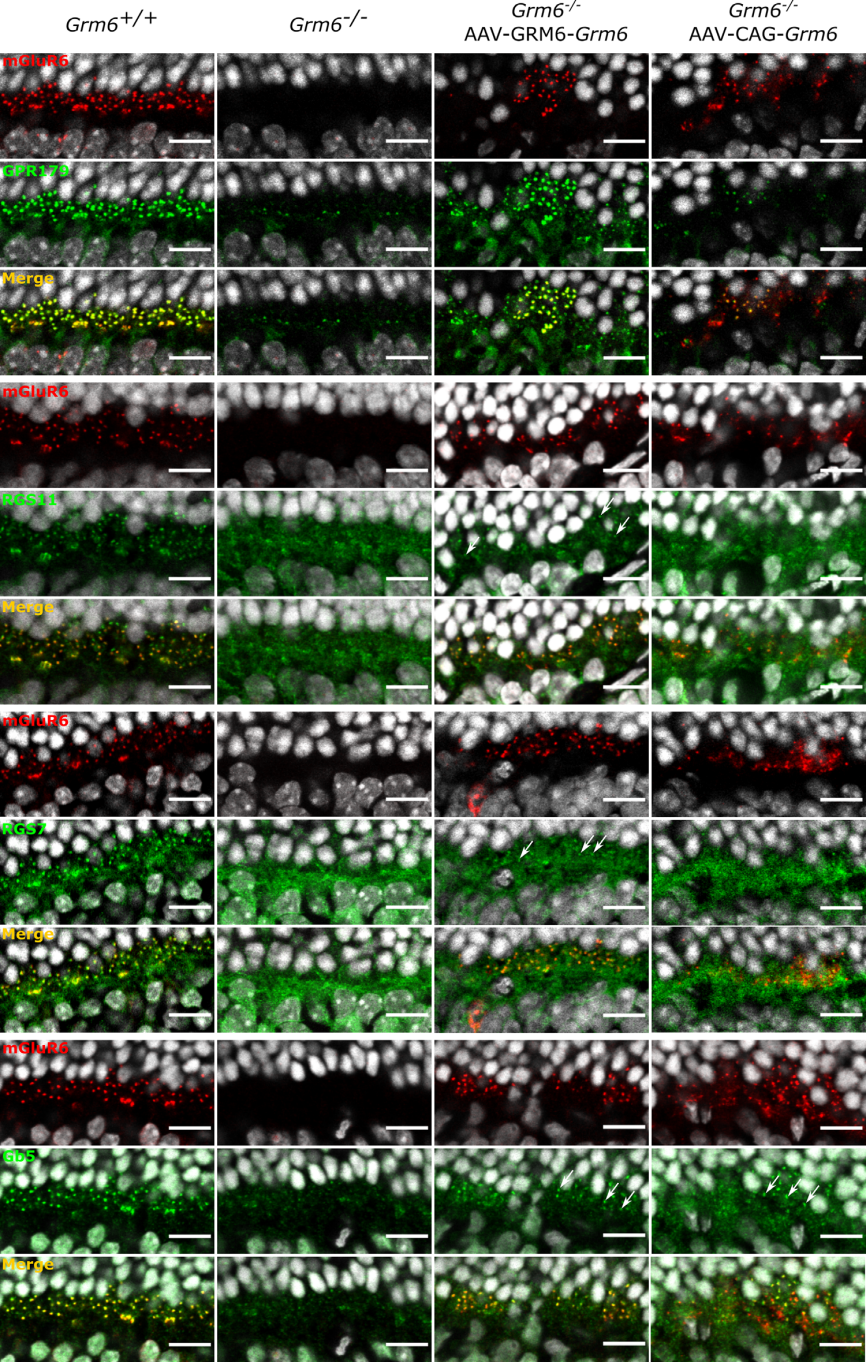
Localization of proteins of the ON-BC signaling cascade after treatment. Representative confocal images of cross-sections centered on the OPL of *Grm6^+/+^*, untreated *Grm6^−^^/^^−^*, *Grm6^−^^/^^−^*-GRM6-*Grm6*, and *Grm6^−^^/^^−^*-CAG-*Grm6* retinas co-stained (*yellow*, merge) with an antibody against mGluR6 (*red*) and against GPR179, RGS11, RGS7, or Gβ5 (*green*). *Arrows* point out the relocalization at the dendritic tips of the ON-BCs. *Scale bar*: 10 µm.

It was previously described that several molecules involved in the signaling cascade of ON-BCs were impacted by the absence of mGluR6.^2^ This is consistent with our findings, which showed reduced or abolished staining of molecules of the same cascade including TRPM1, RGS7, RGS11, and Gβ5 (Figs. 2, 3, second column). Consistent with several observations regarding proper mGluR6-dependant GPR179 localization,^26^^,^^27^ we observed a severe decrease in the localization of GPR179 at the dendritic tips of ON-BCs in *Grm6^−^^/^^−^* mice (Fig. 3). *Grm6^−^^/^^−^*-GRM6-*Grm6* and *Grm6^−^^/^^−^*-CAG-*Grm6* retinas revealed restoration of TRPM1, Gβ5, and GPR179 localization at the dendritic tips of presumably rod BCs after treatment (Figs. 2, 3). In addition, a partial restoration of the localization of RGS7 and RGS11 was noted, which was more obvious in retinas treated with GRM6-*Grm6* than retinas treated with CAG-*Grm6* (Fig. 3). Partial relocalization of the presynaptic protein dystrophin at the presumed rod-to-BC synapse, but not at the cone-to-cone ON-BCs, was obtained in mice retinas treated with GRM6-*Grm6* but was almost absent in mice retinas treated with CAG-*Grm6* (Supplementary Data S1).

### mGluR6 Protein Restoration Did Not Induce a Functional Rescue

**Figure 4.**
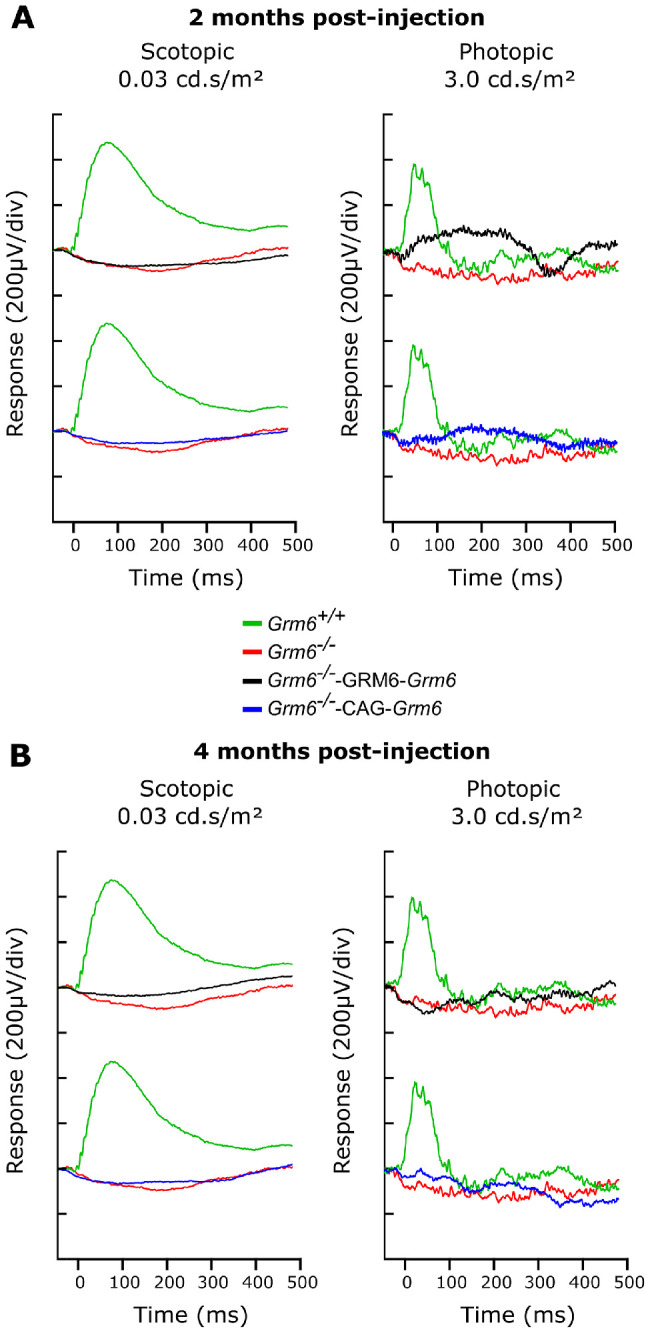
ERG recordings. (**A**) Representative scotopic ERG traces at 2 months post-injection for *Grm6^+^^/^^+^* (*green line*), untreated *Grm6^−^^/^^−^* (*red line*), *Grm6^−^^/^^−^*-GRM6-*Grm6* (black line), and *Grm6^−^^/^^−^*-CAG-*Grm6* (*blue line*) mice at a flash intensity of 0.03 cd·s/m² under scotopic conditions (*left*) and 3.0 cd·s/m² under photopic conditions (*right*). (**B**) Representative scotopic ERG traces at 4 months post-injection for a flash intensity of 0.03 cd·s/m² (*left*) and photopic ERG traces for a flash intensity of 3.0 cd·s/m² (*right*).

Due to disruption of the signal transmission between photoreceptors and ON-BCs in the *Grm6^−^^/^^−^* mouse model, the b-wave was abolished in both scotopic and photopic conditions (Fig. 4). In order to study the functional restoration, ERG recordings were performed on P15-treated mice at two time points: 2 months and 4 months post-injection (*n* = 8). At both time points, none of the treated animals revealed a restoration of the b-wave under either scotopic or photopic conditions after intravitreal injections of either CAG-*Grm6* or GRM6-*Grm6* AAVs (Fig. 4).

## Discussion

GRM6 is the first actor of the signaling cascade at the dendritic tips of ON-BCs, responsible for the transmission of the signal from photoreceptors to ON-BCs. When *GRM6* is mutated it leads to cCSNB. Patients displaying cCSNB have night blindness, high myopia, nystagmus, and sometimes strabismus, all of which can influence the quality of life during the day and at night. The disease can be correctly diagnosed through full-field ERGs. Patients and mice with this gene defect show loss of mGluR6 function and reveal an electronegative ERG waveform in which the a-wave is preserved but the b-wave is absent.^18^^,^^39^ In addition, mice lacking *Grm6* are characterized by the absence of the respective protein, and several other proteins of the same cascade are mislocalized, absent, or reduced at the dendritic tips of ON-BCs. GPR179 and TRPM1 are reduced, and the RGS proteins (Gβ5, RGS7, and RGS11) are absent.^24^ We investigated the localization of these proteins in *Grm6^−^^/^^−^* mice and compared our findings with reported studies. We showed that, in *Grm6^−^^/^^−^* mice, GPR179 localization at the dendritic tips of ON-BCs is dramatically reduced but still present. Ray and co-workers^27^ reported no difference in GPR179 localization in *Grm6^−^^/^^−^* mice in immunolocalization studies, but western blot analysis suggested a decrease in GPR179 of ∼50% in *Grm6^−^^/^^−^* retinas compared to normal retinas. In contrast, Orlandi and co-workers^26^ reported, as we did, a dramatic reduction of GPR179 at the dendritic tips of ON-BCs in a mouse model lacking *Grm6* (*nob3*). In fact, our study and the study by Orlandi et al.^26^ used the same antibody, which differed from that of Ray et al.,^27^ which may explain this discrepancy. Further investigations are needed to explain these different observations. However, GRP179 being reduced but not completely absent at the dendritic tips of ON-BCs in *Grm6^−^^/^^−^* mice indicates that mGluR6 plays a major but not essential role for the proper localization of GPR179 at the dendritic tips.

To date, for most inherited retinal disorders, treatment is unavailable. However, a gene addition approach mediated by AAV for Leber congenital amaurosis was validated by the Food and Drug Administration in the United States 3 years ago, paving the way to treatment of inherited retinal disorders by gene therapy.^40^^–^^43^ CSNB is a rare heterogeneous group of retinal disorders and as yet incurable.^2^ Two gene therapy approaches for cCSNB have been reported so far, one targeting *Nyx*^31^ and the other targeting *Lrit3.*^32^ In both strategies, the wild-type copy of the gene was delivered in newborn (P2 or P5) and adult (P30 or P35) CSNB mice. A partial rescue of the scotopic ERG responses was obtained, mainly in newborn treated mice, under scotopic conditions.^31^^,^^32^ To our knowledge, a gene therapy approach to rescue the phenotype due to the *GRM6*-gene defect is unavailable. Mutations in *GRM6* were found to be the third most prevalent gene defect causing cCSNB.^2^ To rescue the phenotype of mice lacking mGluR6, here we used a combination of the AAV2.7m8 capsid along with either the *GRM6*-200bp/SV40 promoter to drive the expression of *Grm6*^33^ (GRM6-*Grm6*) or a CAG promoter^34^ (CAG-*Grm6*). Although functional restoration in the ERG was not observed, intravitreal injections of adult mice revealed relocalization of mGluR6 and their signaling partners. Furthermore, although the specificity of the GRM6-*Grm6* construct for ON-BCs was greater than that of the CAG-*Grm6* combination, the transduction rate was less than with the non-specific promoter.

Studying the expression of the transgene by RNA in situ hybridization would explain whether the low production of mGluR6 in the treated mice is due to a low transduction efficacy or a problem with ON-BCs producing the protein from the transgene. The lack of functional rescue and relatively low transduction efficiency of our approach may be due to the size and structure of mGluR6. A substantial limitation of AAV vectors is their small packaging capacity, which is generally considered to be <5 kb.^44^ Our constructs were 3.5 kb and 3.8 kb in size for the GRM6-*Grm6* and CAG-*Grm6* constructs, respectively (the coding sequence of *Grm6* is 2.6 kb). Together, these constructs are in the range of <5 kb, so encapsidation of those should not be an issue. Successful gene therapies resulting in partial functional restoration for nyctalopin^31^ and LRIT3^32^ (Varin, J, et al., submitted) using similar vectors have focused on even smaller genes (1.4 kb and 2 kb, respectively), encoding smaller and structurally simpler proteins than mGluR6. We speculate it might be more complicated to produce more complex proteins, such as mGluR6, which has multiple transmembrane domains and more disulfide bonds than smaller molecules. In addition, the constructs used may need to be optimized. Of course, using the full-length promoter and intronic regions to enhance and control expression and protein production would most likely improve the efficacy of the treatment.^45^ However, the size of such a transgene would be too large to be encapsidated by our AAV approach. Oversized AAV vectors encapsidating up to 9 kb of transgene have not been validated for clinical use because gene fragmentation occurs. As an alternative to the oversized AAV approach, various research groups have tested the use of dual and triple AAV vector systems and reported very low levels of protein expression.^46^

Recently, Lu and co-workers^47^ tested different modulations of the *Grm6* promoter in order to increase its efficiency. They reported two promoters with high specificity and higher expression of the transgene than the one we applied by using the endogenous promoter of *Grm6*. The modified sequence was only ∼300 bp larger compared to our GRM6-*Grm6* construct and thus could be easily used for gene therapy delivery to rescue the mGluR6 phenotype. Compared with the construct we used, the fluorescence intensity of mCherry under the control of this new promoter was ∼5 times higher.^47^ However, the number of transduced cells was not significantly improved with this promoter compared to ours. It remains questionable if this promoter would be good enough to increase the transduction efficiency to obtain homogeneous protein immunolocalization and functional rescue. Efficient transduction of ON-BCs is still difficult to obtain, and to overcome this issue other improved AAV–promoter combinations should be tested.

To test the efficiency of those different promoters, fluorescent proteins such as green fluorescent protein or mCherry are often used^33^^,^^47^. However, these promoters used in combination with these genes will localize the respective protein in ON-BCs but not specifically at the dendritic tips of ON-BCs where mGluR6 should be localized for functional rescue^33^^,^^47^. Thus, the transduction efficiency of those vectors may not indicate if trafficking to the dendritic tips of ON-BCs can be achieved. The targeting of transmembrane proteins to neuronal dendrites was previously reported to be myosin dependent.^48^ We tried to improve this trafficking to the dendritic tips by adding a myosin-binding domain to *Grm6*; however, this did not result in further improvement in transduction efficiency nor phenotypic rescue (data not shown). In addition, we waited several months after injection to record ERGs and perform immunolocalization studies, as the peak of transgene expression was shown to be at a maximum 6 to 8 weeks after injection.^49^ However, even 4 months after treatment, the phenotype of treated animals could not be rescued. As the full-field ERG reflects the global response of the retina, it is also possible that the few ON-BCs expressing mGluR6 after treatment were able to depolarize in response to light but, because their numbers were very low, these responses were not detected by ERG. Multi-electrode array recordings or patch-clamp recordings, as described by Scalabrino et al.,^31^ on treated retinas or ON-BC expressing mGluR6 could have addressed this issue, although a major problem is still the transduction rate. Furthermore, optomotor responses could have been used to investigate the functional rescue similarly to the maze test for Leber congenital amaurosis patients treated with AAV-*RPE65*,^41^ but a range of 2.5% to 11% of ON-BCs expressing mGluR6 most likely would not have improved these responses. A subretinal route of delivery could also be considered to enhance the transduction rate of the AAV.^50^

As previously described, proteins involved in cCSNB also have a role in synapse formation and maintenance. Indeed, in *Lrit3^−^^/^^−^* mice, a decreased number of invaginating contacts made by cone ON-BCs at the cone pedicle and a striking decrease in the number of triads^51^ were observed. Normal cone pedicles were also noted, indicating that LRIT3 is not essential for the normal development of the cone-to-cone BC synapse but does play a major role in this process.^51^ The same observation was made in *Gβ5^−^^/^^−^* mice in which the number of triads in the OPL was significantly reduced.^52^ Finally, mGluR6 was shown to play a role at the photoreceptor-to-BC synapse. Indeed, invaginating dendrites of rod BCs are larger and often contain ectopic ribbons, whereas the number of invaginating dendrites of cone ON-BCs and ribbons decreases at the cone pedicles in the *Grm6^−^^/^^−^* mouse model,^29^^,^^53^ and the reduced localization at the OPL of presynaptic proteins such as dystrophin has been described.^30^

In conclusion, there are strong indications that proteins localized at the synapse between photoreceptors and ON-BCs also play a structural role. Interestingly, structural alterations have also been noted in patients^28^. Structural synaptic alterations, or failure to efficiently relocalize presynaptic proteins, could therefore explain the lack of functional rescue despite restoration of mGluR6 in our *Grm6^−^^/^^−^* mice, which were treated at an adult age when the synapses are fully formed. In addition, mGluR6 was also detectable in the rat retina at P6 to P8, diffusely distributed in the somata and dendrites of the INL cells.^54^ This finding may indicate that the presence of mGluR6 may be essential as early as P6 to ensure correct synaptogenesis and functional restoration later. However, with regard to the potential plasticity of the photoreceptors to BC synapses, Wang and colleagues^55^ recently studied the plasticity of the synapses between rods and rod–BCs in a mouse model of retinitis pigmentosa. They showed that in 4-week-old mice a certain plasticity of the synapses could be noticed as treated mice rescued triads of invaginating BC dendrites, and there was more robust and distributed mGluR6 staining in the treated animals. On the other hand, Shen et al.^56^ showed a steep maturational decline of cone-to-cone BC homeostatic plasticity by comparing this plasticity in mice whose cones begin to degenerate at P10 or at P30. Therefore, at least in the case of rod-to-rod BC synapses and potentially in the case of cone-to-cone BC synapses, it appears that P15 retinas are able to remodel the synapse, which could lead to functional rescue. It would be interesting to document if such a plasticity could be obtained in treated cCSNB models through electron microscopy.

Finally, regulatory proteins missing at the dendritic tips of ON-BCs (RGS7, RGS11, and Gβ5) when mGluR6 was absent were detected when mGluR6 was present in the OPL after delivery of the GRM6-*Grm6* construct. This was less obvious after delivery of the CAG-*Grm6* construct, which might partially explain why, even when the transduction rate was increased using a CAG promoter, there was still no functional rescue observed by ERG recordings in these treated mice, as RGS11 and RGS7 are involved in the sensitivity and time course of light-evoked responses^36^.

We showed here for the first time, to our knowledge, the restoration of mGluR6 localization at the dendritic tips of ON-BCs following a gene addition strategy in P15-treated *Grm6^−^^/^^−^* mice. This restoration led to relocalization of other partners of the cascade, such as TRPM1, GPR179, RGS7, RGS11, and Gβ5; however, functional rescue after treatment as measured by ERG failed to appear. Further studies are needed to improve vector–promoter combinations and potentially the mode of administration (and therefore the efficiency of ON-BC transduction), but our findings are encouraging and will serve as a base to develop further gene therapy trials for cCSNB due to mutations in *GRM6*.

## Supplementary Material

Supplement 1
