# Supplementary material for: Restoration of mGluR6 Localization Following AAV-Mediated Delivery in a Mouse Model of Congenital Stationary Night Blindness
Source: Invest Ophthalmol Vis Sci. 2021 Mar 17;62(3):24. doi: 10.1167/iovs.62.3.24 (PMC7980044; doi:10.1167/iovs.62.3.24)
Supplement: Supplement 1 [file iovs-62-3-24_s001.pdf]

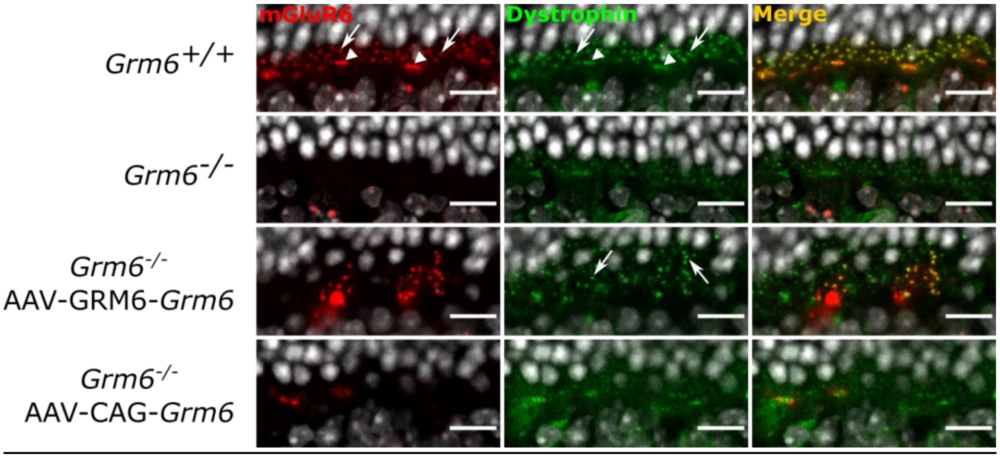

Supplementary data 1: Localization of dystrophin after treatment

Representative confocal images of cross-sections centered on the OPL of *Grm6*$^{+/+}$, untreated *Grm6*$^{-/-}$, *Grm6*$^{-/-}$-GRM6-*Grm6* and *Grm6*$^{-/-}$-CAG-*Grm6* retinas co-stained (yellow, merge) with an antibody against mGluR6 (red) and against dystrophin (green). The presumed rod-to-BC and the cone-to-cone BCs synapses are highlighted with an arrow and arrow head, respectively. Scale bar, 10 µm.
